# Fire Risks at Hospitals

**Published:** 1912-10-12

**Authors:** 


### FIRE RISKS AT HOSPITALS.

There is an old story—we do not vouch for its truth—as to the effects of a fire alarm at a certain large English hospital. Wishing to test the working of the fire drill and regulations, the governing body arranged that a fire alarm should be rung without any warning that no fire really existed. Everything went off with clockwork precision: porters, nurses, students all carried out their allotted duties with disciplined coolness. The patients were quickly transferred to a vacant space at the back of the hospital; those who were bedfast were carried out in their beds just as they lay. But when the test was concluded, and orders were given to remove the beds back again to the wards, it was found that three of the patients had died of fright during their removal from the building.

Now this story, as has been hinted, may or may not be true; but the lesson it teaches is just as patent whether it is true or false. No more appalling disaster from fire can be imagined than that from a big fire at a hospital; and of all hospitals, surely the most dreadful scenes of all would be enacted in a big hospital for children. To depict such horrors would require the graphic pen of a Zola, and it is not necessary to attempt the harrowing task now. But unless the public and local authorities and fire insurance companies can be stirred somehow from their apathy some such catastrophic hecatomb is only too likely some day to startle London.

The Chairman and Vice-Chairman of the Hospital for Sick Children, Great Ormond Street, wrote to the *Times* a year or two ago on the occasion of a fire in a large timber-yard adjacent to their premises. By the good fortune of a wind in a favourable quarter, and by great exertions on the part of the fire brigade and of the hospital resident medical and nursing staff, the hospital was saved from any damage. But the lesson has been forgotten by the public, and Messrs. Lucas and Murray are doing good service by their recent reminder. Their last letter is prompted by the account of a timber-yard blaze at St. Pancras, which spread to neighbouring houses; disaster to the inmates was narrowly avoided.

In a sense, the problem of the London timber-yard is not peculiar to the vicinity of hospitals. Timber-yards which are closely surrounded by dwellings exist in London by scores and hundreds, and they are a serious menace to the lives of those who inhabit such dwellings. In many cases, no doubt, the timber-yard was in existence before the houses were built round it, and it would certainly be hard on the timber merchants to dispossess them because their neighbours chose to build. Like the Great Ormond Street Chairman and Vice-Chairman, we leave it to constituted authorities to find a remedy which shall be efficient yet not unjust. If, after the ventilation which the subject has received, a catastrophe should be attributable to their inaction, there will be no possibility of their shirking the responsibility and the blame. Unfortunately history shows that it required a cholera epidemic to secure the beginnings of sanitation and a Crimean War to reform the War Office, and a knowledge to-day of the same branch of study teaches one that there is no reason to suspect that human heedlessness has in any way been lessened by these classic examples of neglect.

